# Analytical Performance of the Roche LightCycler® *Mycobacterium* Detection Kit for the Diagnosis of Clinically Important Mycobacterial Species

**DOI:** 10.1371/journal.pone.0024789

**Published:** 2011-09-22

**Authors:** Shaheed V. Omar, Andreas Roth, Nazir A. Ismail, Linda Erasmus, Marthie Ehlers, Marleen Kock, Nuraan Paulse, Halima M. Said, Anwar A. Hoosen, Udo Reischl

**Affiliations:** 1 Department of Medical Microbiology, University of Pretoria, Pretoria, South Africa; 2 Labor für Molekulare Diagnostik und Mikrobiologie, HELIOS Klinikum E. v. Behring, Berlin, Germany; 3 National TB Reference Laboratory, National Institute for Communicable Diseases, Johannesburg, South Africa; 4 National Health Laboratory Service, Kimberley, South Africa; 5 Institut für Medizinische Mikrobiologie und Hygiene, Universitätsklinikum Regensburg, Regensburg, Germany; University of Stellenbosch, South Africa

## Abstract

**Background:**

The LightCycler® Mycobacterium Detection Kit based on real-time PCR technology for the detection of *Mycobacterium tuberculosis*, *Mycobacterium avium* and *Mycobacterium kansasii* was recently developed. This study evaluated its analytical sensitivity, specificity and reproducibility.

**Methodology/Principal Findings:**

Plasmid standards were prepared and used to determine the limit of detection. The assay was also performed against organisms other than mycobacteria, other mycobacterial strains and interfering substances to exclude cross-reactivity and interference. Reference standards were prepared and tested to assess the assay's reproducibility. All PCR assays were performed using the LightCycler® 2.0 Instrument. The detection limit for *M. tuberculosis* was 28 copies per microlitre. Neither cross-reactivity nor interference occurred with non-mycobacterial organisms and substances tested. Overall reproducibility for consecutive measurements, run-to-run, lot-to-lot, day-to-day and laboratory-to-laboratory achieved a coefficient of variance of less than two percent.

**Significance:**

The LightCycler® *Mycobacterium* Detection kit has shown to be a robust and accurate assay with the potential to be used as a rapid TB diagnostic test.

## Introduction

In 2009, 5.8 million cases of tuberculosis were reported to the national tuberculosis control programmes globally where only 57% of the globally notified pulmonary cases were smear positive. Furthermore, the World Health Organization estimates that close to 1.3 million people die as a consequence of the disease. Therefore serious efforts are needed to strengthen the control of tuberculosis [1].

The occurrence of nontuberculous mycobacteria (NTM) from pulmonary samples is occurring with greater frequency driven mainly by the high rates of people living with HIV [2]. The commonest NTM's associated with pulmonary infection are *M. avium* complex, *M. kansasii*, *M. abscessus* and *M. fortuitum* [2]–[4].

Since tuberculosis is a disease for which the vaccine has failed to provide complete protective immunity [5], its control has relied on early diagnosis and treatment. However, smear microscopy which is the primary tool has a low sensitivity and culture has a very lengthy turnaround time despite its sensitivity [6]. Several nucleic acid amplification tests are available and all aimed at improving the diagnosis of tuberculosis [6]–[9]. However, none of these assays have performed adequately to become the new standard for diagnosis. Recently the Xpert® MTB/Rif (Cepheid, USA) test, based on real-time polymerase chain reaction (PCR), has received WHO endorsement [10].

Real-time PCR provides great hope as it is a highly sensitive, specific, and rapid molecular method. In addition, an assessment of the dissociation-characteristics of double-stranded DNA during heating known as melting curve analysis can provide species identification [11]–[13]. The LightCycler® System (Roche Diagnostics, Germany) has been widely used as a platform for real-time PCR. This platform has the advantage of a high-throughput capacity which is important in a high-burden setting.

A new commercial molecular diagnostic assay the LightCycler® Mycobacterium Detection Kit (Roche Products (Pty) Ltd, Randburg, South Africa), was developed for use on respiratory samples. It is based on the real-time PCR technology for the detection of *M. tuberculosis*, *M. avium* and *M. kansasii* within 90 minutes and amplifies the 16s ribosomal RNA (rRNA) gene which includes the hypervariable region A. The products are detected using the fluorogenic hybridization probes and species identification by melting curve analysis on the LightCycler® 2.0 (Roche Diagnostics, Germany). A synthetic internal control has been integrated into the reaction. This study evaluated the assay's analytical sensitivity, specificity and reproducibility.

## Methods

This study was performed at 4 centres viz. the HELIOS Klinikum E. v. Behring (Germany), Institute of Medical Microbiology Hygiene - University of Regensburg (Germany), Department of Medical Microbiology - University of Pretoria (South Africa) and the National Health Laboratory Service - Kimberley laboratory (South Africa).

These centres were involved in analytically evaluating the LightCycler® Mycobacterium Detection Kit by preparation of plasmid standards to determine the limit of detection, a challenge panel consisting of other mycobacterial species, non-mycobacterial organisms and interfering substances to assess specificity and mycobacterial standards to measure reproducibility. The assay's reproducibility in terms of consecutive measurements (intra-run), run-to-run, day-to-day, lot-to-lot and laboratory-to-laboratory was performed at the two South African centres only.

### DNA extraction

Mycobacterial reference standards and simulated respiratory samples were decontaminated using N-acetyl-L-cysteine-Sodium hydroxide (NaLc-NaOH) [14] and resuspended in 1.5 ml of phosphate buffer containing 0.5% Tween 80 which allowed better homogenization and even distribution of the bacilli within the suspension prior to extraction.

The COBAS® Amplicor Respiratory Sample Preparation Kit (Roche Diagnostics, Germany) was used according to the manufacturer's instructions. Briefly, a 100 µl aliquot of the NALC-NaOH decontaminated sample was mixed with the kit's wash solution and centrifuged (14,000× g for 10 min). After centrifugation, the supernatant was removed and the lysis reagent was added to the pellet. After brief vortexing, the suspension was incubated at 60°C for 45 min to complete lysis of the mycobacteria. Thereafter the neutralizing reagent was added making a total volume of 200 µl neutralized DNA sample. Four microlitres (4 µl) of the neutralized sample was used as template DNA for subsequent PCR testing.

### PCR assay

All PCR assays were performed using the LightCycler® 2.0 (Roche, Germany) real-time instrument. For amplification and detection, the LightCycler® Mycobacterium Detection Kit (Roche Diagnostics) was used according to the manufacturer's instructions. Samples were amplified as follows: initial denaturation step at 95°C for 10 min to activate the FastStart *Taq* DNA polymerase and 45 cycles of denaturation at 95°C for 10 sec, annealing at 50°C for 10 sec, and an extension at 72°C for 20 sec.

Following the amplification phase, a melting curve analysis at 40°C to 65°C was performed with a temperature transition rate of 0.1°C/sec to determine the melting temperature (T_m_) values for the sequences targeted by the hybridization probes. T_m_ values were either automatically or manually assigned from a plot generated by the instrument. A T_m_ of 53.5–56.5°C indicated *M. tuberculosis*, 48–51°C *M. avium* and 57–60°C *M. kansasii*.

The generation of target amplicons for each sample was monitored between the annealing and the elongation steps at 640 nm. Samples positive for the specific amplicon (an approximate 200 bp fragment of the mycobacterial 16S rDNA) were identified by the instrument at the cycle number where the fluorescence attributable to the target sequences exceeded that measured for background. Samples scored as positive by the instrument were confirmed by visual inspection of the amplification curve (cycle number *versus* fluorescence value) generated by the instrument.

Analysis was performed after activation of the colour compensation using the following steps: a positive control showing an exponential amplification curve with a defined crossing point (CP) value of ∼26.5 with a signal intensity of >0.1; a positive control showing a melting curve T_m_ value of ∼57–60°C for *M. kansasii* with a signal intensity of >0.1, a negative control showing an exponential amplification curve for the internal control only (detection channel 705/back 530 nm only) with a defined CP value of ∼30–34 with a signal intensity of >0.1. Samples were regarded positive for mycobacteria if they had exponential amplification with a CP value of less than 35 with a signal intensity of >0.02 and negative with no amplification on the 640/back 530 nm channel and exponential amplification for the internal control in the 705/back 530 nm channel having a signal intensity of >0.02.

### Preparation of Mycobacterial Reference Standards

*M. tuberculosis*, *M. avium* and *M. kansasii* clinical isolates were cultured on 7H11 agar (Becton Dickinson, USA) for 2 weeks. Homogenization of harvested bacteria was achieved by vortexing with glass beads (2 mm) for 30 seconds followed by subsequent sub-cultivation in 7H9 broth (Becton Dickinson, USA). Once culture broths reached the logarithmic growth phase corresponding to a McFarland 0.5 turbidity the broth was removed and dilution standards prepared. The dilutions were further homogenized by vortexing with glass beads. These bacterial suspensions contained approximately 10^6^ colony forming units per millilitre (CFU/ml).

### Preparation of simulated respiratory samples

Samples of bronchoalveolar lavage (BAL) originating from patients without any clinical signs or symptoms of tuberculosis or other mycobacterial infections were used as template material for preparation of the simulated respiratory samples for the reproducibility study. The BAL samples were spiked with the prepared mycobacterial reference standards. Culture of the spiked samples yielded 13 000 CFU/µl for *M. tuberculosis*, 39 000 CFU/µl for *M. avium* and 30 000 CFU/µl for *M. kansasii*. These stock solutions were used to prepare the dilution series.

### Analytical Sensitivity (Limit of Detection)

Plasmid standards using a single gene copy were used to determine the Limit of Detection (LOD) of the assay. Plasmid standards for *M. tuberculosis* were tested in a 6 fold dilution series: 130, 65, 33, 16, 7 and 2 copies per microlitre. All measurements were performed in 20-fold replicates (5 replicates in 4 independent runs on the same LightCycler® Instrument and the same day). For verification of the measurements the reference standards were run in parallel on the FDA approved Cobas® Amplicor *Mycobacterium* Tuberculosis Test. The effect of interfering substances (table 1) on PCR inhibition was also assessed.

**Table 1:** Challenge organisms and interfering substances used to determine the analytical sensitivity and specificity of the test.

Organisms other than Mycobacteria	Mycobacterial organisms	Interfering substances
Adenovirus DNA	*M. peregrinum*	Haemoglobin
HIV RNA	*M. marinum*	Ethambutol
Influenza A virus RNA	*M. gordonae*	Clarithromycin
Parainfluenza RNA	*M. flavescens*	Azithromycin
*Aspergillus fumigatus*	*M. engbaekii*	Isoniazid
*Candida albicans*	*M. smegmatis*	Rifampicin
*Neisseria meningitidis*	*M. neoaurum*	Pyrazinamid
*Haemophilus inluenzae*	*M. celatum*	
*Moraxella catarhalis*	*M. fortuitum*	
*Moraxella osloensis*	*M. ratisbonense*	
*Bordetella pertussis*	*M. kansasii*	
*Escherichia coli*	*M. simiae*	
*Enterobacter cloacae*	*M. malmoense*	
*Klebsiella pneumoniae*	*M. szulgai*	
*Chlamydia trachomatis*	*M. hasiaticum*	
*Salmonella typhi*	*M. bovis/H37*	
*Proteus vulgaris*	*M. terrae*	
*Legionella pneumphila*	*M. shimoidei*	
*Acinetobacter baumanii*	*M. xenopi*	
*Pseudomonas aeruginosa*	*M. phlei*	
*Helicobacter pylori*	*M. intracellulare*	
*Nocardia asteroides*	*M. scrofulaceum*	
*Corynebacterium diphteria*	*M. avium*	
*Staphylococcus aureus*	*M. chimerae*	
*Legionella micdadei*	*M. elephantis*	
*Streptococcus Group C*		
*Streptococcus Gruppe F*		
*Streptococcus pneumoniae*		
*Mycoplasma pneumoniae*		

### Analytical specificity

In order to check for cross reactivity, a challenge panel was prepared which consisted of other mycobacterial species and non-mycobacterial organisms (table 1); this was tested in duplicate using the LightCycler® *Mycobacterium* Detection Kit. The concentrations of the template nucleic acids were determined by photometric measurement and quantitative *in-house* PCR assays [15]. The species identities of all bacterial and fungal strains were verified by complete 16S rDNA sequencing.

### Reproducibility Study

The reproducibility study was designed to measure the variability of the T_m_ and CP measurements of the assay. For this reproducibility study the following standards were used *M. tuberculosis* at concentrations of 1625 cp/µl, 325 cp/µl, 65 cp/µl and 13 cp/µl, *M. avium* at 4875 cp/µl, 487.5 cp/µl and 48.75 cp/µl, and *M. kansasii* concentrations of 3750 cp/µl, 375 cp/µl and 37.5 cp/µl. In each run, these standards were tested in triplicate for *M. tuberculosis* and duplicate for both *M. avium* and *M. kansasii*. The study was performed at two laboratories; each using a single technician, with a total of 60 runs completed over a 10 day period. The reproducibility of consecutive measurements (intra-run), run-to-run, day-to day, lot-to-lot and laboratory-to-laboratory was measured. A coefficient of variance (CV) of less than ten percent was expected.

### Statistical analysis

The limit of detection (LOD) was determined by using the Probit method, where, the number of positive responses were converted to a ratio for each of the test concentrations and fitted to a regression model of binary response variables. The LOD was read off the generated graph at the 95% probability for response. For the determination of the measurement precision, the run-to-run, day-to-day, lot-to-lot and laboratory-to-laboratory variation, a variance component analysis for the crossing points (CP) and melting temperatures (Tm) of *M. tuberculosis, M. avium and M. kansasii* at different concentrations was carried out. The variance component analysis was performed. All statistical analyses were carried out using the statistical software package JMP 7.0 (SAS/STAT® Software).

## Results

### Analytical Sensitivity (LOD)

**Figure 1:**
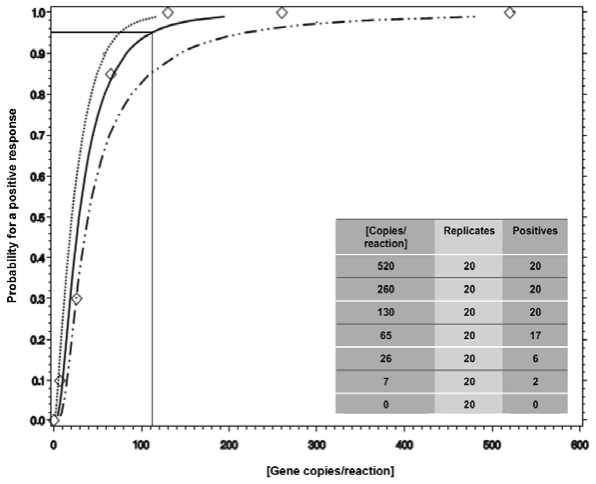
Probit analysis for determination of the Limit of Detection.

Plasmid standards with the target gene were used to determine the limit of detection of the assay. Based on Probit analysis the LOD at the 0.95 (95%) probability for response was calculated at 28 copies per µl [95%CI: 19, 54] or 112 copies per reaction [95%CI: 76, 216] (Figure 1). Furthermore, interfering substances had no effect on PCR performance.

### Analytical Specificity

For all non mycobacterial organism tested the specific primer and probe set of the LightCycler® *Mycobacterium* Detection Kit showed no cross-reactivity with non-mycobacterial organisms. However, two other mycobacterial species were detected *M. flavescens* with a T_m_ around 50°C (expected for *M. avium*) and *M. scrofulaceum* appeared with a melting point around 58°C (expected for *M. kansasii*) and no cross-reactivity in the melting temperature range for *M. tuberculosis*.

### Reproducibility

The coefficient of variance for the CP and T_m_ values of *M. tuberculosis, M. avium and M. kansasii* at different concentrations were calculated. The T_m_ values for the run-to-run, day-to-day and lot-to-lot had a coefficient of variance (CV) of less than 2%. The majority of samples demonstrated CV's below 0.4% (Table 2) with a standard deviation of between 0.1–0.2°C. The highest variance for the T_m_ of *M. tuberculosis* and *M. avium* occurred in the laboratory-to-laboratory component due to three outliers at the Pretoria site from a total of 760 measurements.

**Table 2:** Variance component analysis of the Crossing Point values and Melting temperature range.

CROSSING POINTS					
Reference Standard	Intra Run Variability	Run to Run Variability	Day to Day Variability	Lot to Lot Variability	Lab to Lab Variability
	Repeatability	Intermediate Precision			Reproducibility
***M. tuberculosis*** ** (325 cp/µl)**	SD: 0.371	SD: 0.303	SD: 0.102	SD: 0.003	SD: 0.180
	CV: 1.21%	CV: 0.99%	CV: 0.33%	CV: 0.01%	CV: 0.59%
***M. tuberculosis*** ** (1625 cp/µl)**	SD: 0.264	SD: 0.0	SD: 0.071	SD: 0.0	SD: 0.067
	CV: 0.94%	CV: 0.0%	CV: 0.25%	CV: 0.0%	CV: 0.24%
***M. avium*** ** (488 cp/µl)**	SD: 0.314	SD: 0.129	SD: 0.149	SD: 0.0	SD: 0.106
	CV: 1.02%	CV: 0.42%	CV: 0.48%	CV: 0.0%	CV: 0.34%
***M. avium*** ** (4875 cp/µl)**	SD: 0.290	SD: 0.0	SD: 0.094	SD: 0.110	SD: 0.0
	CV: 1.03%	CV: 0.0%	CV: 0.33%	CV: 0.4%	CV: 0.0%
***M. kansasii*** ** (375 cp/µl)**	SD: 0.275	SD: 0.125	SD: 0.120	SD: 0.108	SD: 0.070
	CV: 0.88%	CV: 0.4%	CV: 0.39%	CV: 0.3%	CV: 0.22%
***M. kansasii*** ** (3750 cp/µl)**	SD: 0.214	SD: 0.090	SD: 0.148	SD: 0.0	SD: 0.0
	CV: 0.74%	CV: 0.31%	CV: 0.51%	CV: 0.0%	CV: 0.0%

MELTING TEMPERATURE					
Reference Standard	Intra Run Variability	Run to Run Variability	Day to Day Variability	Lot to Lot Variability	Lab to Lab Variability
	Repeatability	Intermediate Precision			Reproducibility
***M. tuberculosis*** ** (325 cp/µl)**	SD: 0.077	SD: 0.079	SD: 0.047	SD: 0.085	SD: 0.174
	CV: 0.14%	CV: 0.14%	CV: 0.08%	CV: 0.15%	CV: 0.31%
***M. tuberculosis*** ** (1625 cp/µl)**	SD: 0.070	SD: 0.089	SD: 0.0	SD: 0.088	SD: 0.150
	CV: 0.12%	CV: 0.16%	CV: 0.0%	CV: 0.2%	CV: 0.27%
***M. avium*** ** (488 cp/µl)**	SD: 0.061	SD: 0.085	SD: 0.0	SD: 0.050	SD: 0.168
	CV: 0.12%	CV: 0.17%	CV: 0.0%	CV: 0.1%	CV: 0.34%
***M. avium*** ** (4875 cp/µl)**	SD: 0.065	SD: 0.087	SD: 0.0	SD: 0.030	SD: 0.173
	CV: 0.13%	CV: 0.18%	CV: 0.0%	CV: 0.06%	CV: 0.35%
***M. kansasii*** ** (375 cp/µl)**	SD: 0.091	SD: 0.088	SD: 0.0	SD: 0.090	SD: 0.219
	CV: 0.16%	CV: 0.15%	CV: 0.0%	CV: 0.2%	CV: 0.37%
***M. kansasii*** ** (3750 cp/µl)**	SD: 0.104	SD: 0.076	SD: 0.0	SD: 0.100	SD: 0.212
	CV: 0.18/%	CV: 0.13%	CV: 0.0%	CV: 0.2%	CV: 0.36%

Key: cp/µl: copies/microlitre SD: standard deviation and CV: coefficient of variance.

**Figure 2:**
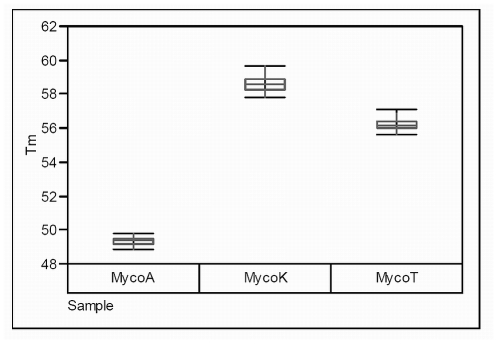
Pooled Melting Temperature (Tm) range of the *M. tuberculosis* (MycoT), *M. avium* (MycoA) and *M. kansasii* (MycoK).

There was a variance in the mean T_m_ of approximately 0.3°C–0.4°C across all the mycobacterial concentrations between the Pretoria and Kimberley sites (Table 3). The standard deviation for the T_m_ ranged between 0.1–0.2°C and these were independent of sample concentrations. However, in a single measurement from the 900 tested a T_m_ for *M. tuberculosis* occurred in the specified melting temperature range of *M. kansasii* (57–60°C) (Figure 2).

**Table 3:** Mean and Standard Deviations for the different concentrations of the three Mycobacteria.

PRETORIA					
Sample	Concentration [copies/µl]	Mean CP	Standard Deviation CP	Mean Tm	Standard Deviation Tm
*M. tuberculosis*	13	31.65	0.471	56.59	0.260
*M. tuberculosis*	65	31.55	0.357	56.45	0.128
*M. tuberculosis*	325	30.40	0.307	56.26	0.129
*M. tuberculosis*	1625	27.97	0.260	56.22	0.107
*M. avium*	48.75	31.80	1.087	49.56	0.126
*M. avium*	487.5	30.68	0.320	49.48	0.103
*M. avium*	4875	28.23	0.280	49.43	0.122
*M. kansasii*	37.5	32.13	0.406	59.41	0.174
*M. kansasii*	375	31.06	0.277	58.65	0.135
*M. kansasii*	3750	28.87	0.265	58.49	0.148

KIMBERLEY					
Sample	Concentration [copies/µl]	Mean CP	Standard Deviation CP	Mean Tm	Standard Deviation Tm
*M. tuberculosis*	13	32.60	0.715	56.21	0.193
*M. tuberculosis*	65	32.09	0.462	56.07	0.131
*M. tuberculosis*	325	30.72	0.480	55.95	0.131
*M. tuberculosis*	1625	28.17	0.247	55.95	0.124
*M. avium*	48.75	32.37	0.948	49.25	0.141
*M. avium*	487.5	30.88	0.402	49.19	0.126
*M. avium*	4875	28.28	0.287	49.15	0.110
*M. kansasii*	37.5	32.88	0.478	58.96	0.165
*M. kansasii*	375	31.29	0.356	58.27	0.133
*M. kansasii*	3750	28.84	0.283	58.11	0.138

CP = crossing point TM = melt temperature.

The mean and standard deviation (SD) for the CP values were dependent on the concentration of organisms within the samples (table 3). At the higher concentrations the variance ranged from 0.22–1.02% with a SD range of 0.247–0.480 and at the lower concentrations the variance ranged from 0.0–1.03% with a SD range of 0.357–1.087 (table 2 & 3).

## Discussion

Improved diagnostic tests for tuberculosis are urgently needed and this has been demonstrated in several studies [8], [16]. Diagnostics with better sensitivity and the ability to differentiate between *M. tuberculosis* and non-tuberculous mycobacteria are required. The application of nucleic acid amplification techniques for tuberculosis diagnosis is promising as it can be done directly on patient specimens with corresponding high sensitivity/specificity and a rapid turnaround time. Real-time PCR provides an advanced methodology due to its unique ability to monitor the complete DNA amplification process and identify specific products within the same reaction using fluorescence techniques. The initial high cost of this technology has decreased considerably with the continual improvement in their capability and accuracy [17].

This study showed the LightCycler® *Mycobacterium* Detection kit to be a robust and accurate assay. The limit of detection as low as 28 copies per microlitre (112 copies/reaction) is promising when compared with other TB molecular assays although not as sensitive as the Xpert® MTB/Rif assay (Cepheid, USA) [18]–[20]. The assay has the potential of being used as a rapid TB diagnostic in place of smear microscopy where resources are available. The lack of sensitivity in comparison to the Xpert® MTB/Rif assay may be due to the initial specimen volume of a 100 µl in comparison to the 1.5 ml used by the Cepheid system [20]. Studies to evaluate changes in the input volume to improve test performance are required.

This study was reproducible as it showed good performance at two independent sites an academic laboratory and a routine diagnostic laboratory in South Africa. The overall accuracy and precision for the run to run, laboratory to laboratory, lot to lot, day to day and intra-assay variance exceeded expected outcomes with variability as low as 1% in the medium to high concentration samples. The higher mean melting temperature range (∼0.3–0.4°C) observed in the Kimberley Laboratory when compared to Pretoria could be attributed to the instruments or other environmental parameters. It was also noted that in a single sample from the 900 tested the T_m_ value for *M. tuberculosis* in the Kimberley Laboratory overlapped the lower specified T_m_ value for *M. kansasii* range. Adjustment of the *M. kansasii* T_m_ range to accommodate this finding is recommended.

The assay did not cross react with any of the non-mycobacterial organisms tested nor was the assay inhibited by the tested interfering substances. All but two of the tested mycobacterial species cross-reacted within the T_m_ range of *M. avium* and *M. kansasii*, however, no cross-reactivity occurred within the T_m_ range for *M. tuberculosis*. The two mycobacterial species detected by the challenge panel were *M. scrofulaceum* which is usually isolated from lymph nodes and *M. flavescens* which has been rarely isolated in humans [21]. The LightCycler® *Mycobacterium* Detection Kit proved to be specific for *M. tuberculosis*, *M. avium* and *M. kansasii* which are clinically important for respiratory infections especially in TB/HIV co-infection settings. The test can further be improved to detect other mycobacterial species based on their unique melting temperatures. This would have a direct impact on the early and correct management of the affected patients.

The *LightCycler® Mycobacterium Detection kit* showed minimal variance when applied using different operators and environments highlighting its robustness. The overall, analytical performance make this kit a candidate tool in the raoid diagnosis of tuberculosis. Clinical evaluations in routine settings are required to validate its performance.
